# Facilitators and barriers of appropriate and timely initiation of intravenous fluids in patients with sepsis in emergency departments: a consensus development Delphi study

**DOI:** 10.1186/s12912-023-01561-w

**Published:** 2023-10-27

**Authors:** Gladis Kabil, Steven A. Frost, Deborah Hatcher, Amith Shetty, Stephen McNally

**Affiliations:** 1 Western Sydney University, School of Nursing and Midwifery, Sydney, Australia; 2 Department of Emergency, Westmead Hospital, Sydney, Australia; 3 South Western Sydney Nursing and Midwifery Research, Ingham Institute of Applied Medical Research, Sydney, Australia; 4 University of New South Wales, Sydney, Australia; 5 School of Nursing and Midwifery, University of Wollongong, Wollongong, Australia; 6 Westmead Institute for Medical Research, Sydney, Australia; 7 NSW Ministry of Health, Sydney, Australia

## Abstract

**Background:**

Sepsis is a life-threatening medical emergency in which appropriate and timely administration of intravenous fluids to patients with features of hypotension is critical to prevent multi-organ failure and subsequent death. However, compliance with recommended fluid administration is reported to be poor. There is a lack of consensus among emergency clinicians on some of the determinant factors influencing fluid administration in sepsis. Thus, the aim of this study was to identify the level of consensus among key stakeholders in emergency departments regarding the facilitators, barriers, and strategies to improve fluid administration.

**Methods:**

The modified Delphi questionnaire with 23 statements exploring barriers, facilitators, and strategies to improve fluid administration was developed from the integration of findings from previous phases of the study involving emergency department clinicians. A two-round modified Delphi survey was conducted among key stakeholders with managerial, educational, supervision and leadership responsibilities using a “Reactive Delphi technique” from March 2023 to June 2023. The statements were rated for importance on a 9-point Likert scale. The RAND/UCLA Appropriateness Method (RAM) was used to identify the level of consensus (agreement/disagreement).

**Results:**

Of the 21 panellists who completed Round 1 survey, 18 (86%) also completed Round 2. The panellists rated 9 out of 10 (90%) barriers, 3 out of 4 (75%) facilitators and all 9 (100%) improvement strategies as important. Out of the total 23 statements, 18 (78%) had agreement among the panellists. Incomplete vital signs at triage (Median = 9, IQR 7.25 to 9.00) as a barrier, awareness of importance of fluid administration in sepsis (Median = 9, IQR 8.00 to 9.00) as facilitator and provision of nurse-initiated intravenous fluids (Median = 9, IQR 8.00 to 9.00) as an improvement strategy were the highest rated statements.

**Conclusion:**

This is the first Delphi study identifying consensus on facilitators, barriers, and strategies to specifically improve intravenous fluid administration in sepsis in Australia. We identified 18 consensus-based factors associated with appropriate and timely administration of intravenous fluids in sepsis. This study offers empirical evidence to support the implementation of the identified strategies to improve patient outcomes.

**Supplementary Information:**

The online version contains supplementary material available at 10.1186/s12912-023-01561-w.

## Background

Sepsis, a dysregulated host immune response to infection resulting in organ dysfunction is one of the leading causes of death across the world. Causing over 5.3 million deaths per year, sepsis is recognised as a global health priority [1]. A key determinant of mortality is tissue hypoperfusion. In order to restore cardiac output, the Surviving Sepsis Campaign recommends administration of at least 30mL/kg of intravenous fluids within the first three hours of recognition of patients with features of hypoperfusion, administration of empirical antibiotics and obtaining blood cultures and measuring serum lactate [2]. Implementation of these guidelines have resulted in an overall 16.7% decrease in mortality over the last decade [3]. However, recent trends show sepsis related mortality in Australia still remains high and about 12% of patients admitted with sepsis die, which is 10.9 times higher than non-sepsis patients [4]. Despite the launch of prominent sepsis awareness programs such as the “Sepsis Kills” in NSW, our previous study [5] shows that the compliance with intravenous fluid administration remains poor and the factors influencing fluid administration are complex [5].

This modified Delphi study is the final stage from a larger mixed-method study in which quantitative data was obtained from retrospective chart reviews [5] and qualitative data was obtained from focus groups [6] among emergency nurses and physicians who are directly involved in care of patients from the four Australian emergency departments. The quantitative and qualitative findings were then integrated using an implementation framework [7] (Integrated findings manuscript currently under review). The primary goal for this modified Delphi study is to determine consensus related to facilitators, barriers and strategies for improvement associated with appropriate and timely intravenous fluid administration for patients with sepsis in emergency departments.

The Consolidated Framework for Implementation Research (CFIR) was used to comprehensively elicit the factors associated with effective implementation. The CFIR was developed by Damschroder et al. [7] and adapted for use in this study. It is a determinant framework that helps with the identification and explanation of factors that influence clinical practice change. CFIR not only focuses on factors influencing the behaviour of an individual healthcare provider such as delays in initiating initial intravenous fluid in patients with sepsis but also considers broader organisational and societal factors that may influence practice [7]. According to the CFIR, for intervention strategies to be effective, they have to be tailored specifically to relevant positive (facilitators) and negative (barrriers) determinants of clinical practice. In addition, one of the key domains of CFIR proposes that in the “Inner Setting”, there should be “Readiness for Implementation” and “Leadership Engagement” while recognising the facilitators and barriers and identifying future strategies to improve practice. This managerial support is critical for the future implementation to be succesful.

The qualitative phase [6] confirmed a lack of consensus among the emergency clinicians with some of the perceived facilitators and barriers of appropriate and timely initiation of intravenous fluids in sepsis. As a pre-implementation study, it is important to identify actionable findings that can be used to design interventions to improve fluid administration in sepsis. The Delphi technique is a popular method which seeks to identify consensus on the opinion of ‘experts’ through a series of structured survey rounds [8].

In this study, we aimed to identify the level of consensus among the key stakeholders with management, leadership, supervision, and educational roles in emergency departments regarding the facilitators, barriers and strategies identified in the previous phases through a modified Delphi study. This study also helps with rating the importance of each identified facilitator, barrier, and strategy so interventions can be prioritised based on consensus and agreed level of importance. While designing interventions, we need to consider the limited healthcare resources and increasing demand for them. Choosing strategies that are agreed upon, will enable these resources to be used optimally.

## Methods

This study was guided by the Delphi survey guidelines [9] and the RAND/UCLA Appropriateness Method (RAM) [10] to identify consensus among the stakeholder panellists. This modified Delphi study was conducted in two rounds of electronic surveys. Reactive Delphi technique [11] was used where the panellists ‘reacted’ to previously prepared statements from integrated findings of the previous phases of the study rather than to generate a new list of items. The Lincoln and Guba principles [12] of credibility (safety in numbers in Delphi where several panellists are less likely to arrive at an incorrect conclusion compared to single individuals); fittingness (conclusions are strengthened by reasoned argument); and confirmability (use of expert panellists who are key stakeholders in emergency departments increasing the validity) as provided in the Delphi guidelines [9] were followed. Ethical approval was received from the Western Sydney Local Health District Human Research Ethics Committee (HREC2022/ETH00374), and data were collected between March 2023 and June 2023. The anonymity of the panellists was maintained throughout the study. The panellists (emergency nurses and physicians with direct managerial or leadership or supervision or educational roles) were asked to:

1. Rate the previously prepared statements according to their importance based on their perspectives,
2. Suggest additional facilitators, barriers and strategies not included in the statements,
3. Provide reasons for their disagreement on statements if any.

### The questionnaire

The questionnaire (Appendix 1) was created in REDCap (Research Electronic Data Capture) tool [13, 14] using the meta-inferences from the integrated findings (Integrated findings manuscript currently under review) of the previous phases of the study [5, 6] reported elsewhere. The questionnaire consists of three dimensions and a total of 23 statements [i] barriers (10 statements); [ii] facilitators (4 statements) and [iii] strategies to improve practice (9 statements). These statements refer to the factors identified in the previous phases of the study and therefore the terms statements and factors are used interchangeably in this paper. The instrument was pretested on a convenience sample of five expert emergency nurses, physicians, and researchers with emergency nursing background. Responses from the pre-test were not included in the analysis as the expert panel did not meet the criteria for key stakeholder panellists.

In Round 1, the panellists received a demographic questionnaire and option to include free text comments regarding any additional barriers, facilitators, or strategies they would like to include. The free text comments option was provided to identify the higher order cognitive processes that occur while the panellists make decisions about ranking the statements in electronic surveys [15]. These comments were provided unaltered (anonymised and minor editorial changes made) to panellists in Round 2 to ensure fair representation of the data as per the Delphi Guidelines [9].

### Panellist selection

Key stakeholders among emergency nurses and physicians with a direct role in management, leadership, education, or supervision such as heads of departments, senior staff specialists, nurse managers, nurse unit managers and educators were identified using a purposive sampling technique. The investigator sent email invitations to 23 identified panellists from the four participating sites. A brief orientation of the findings from the previous phases of the study was provided to those who consented to participate. This orientation enabled the panellists to familiarise themselves with the context of the study [16].

### Data collection

**Fig. 1:**
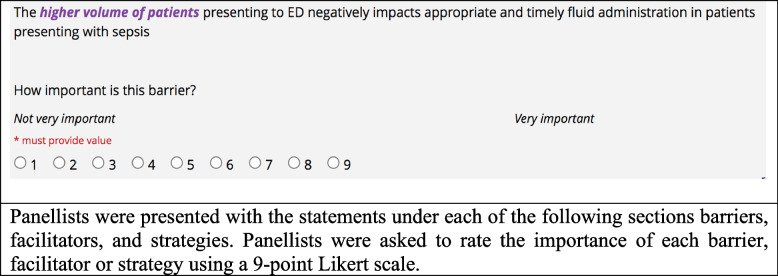
Round 1 questions

 In Round 1, panellists used 9-point Likert scales to rate the importance of statements referring to the barriers, facilitators, and strategies to improve timely fluid administration from their perspective (Fig. 1). We requested panellists to provide free-text comments including any additional facilitators, barriers, and strategies that they felt had not been included in the statements. Within 3 weeks of Round 1 completion, panellists were contacted to participate in the Round 2 survey.

**Fig. 2:**
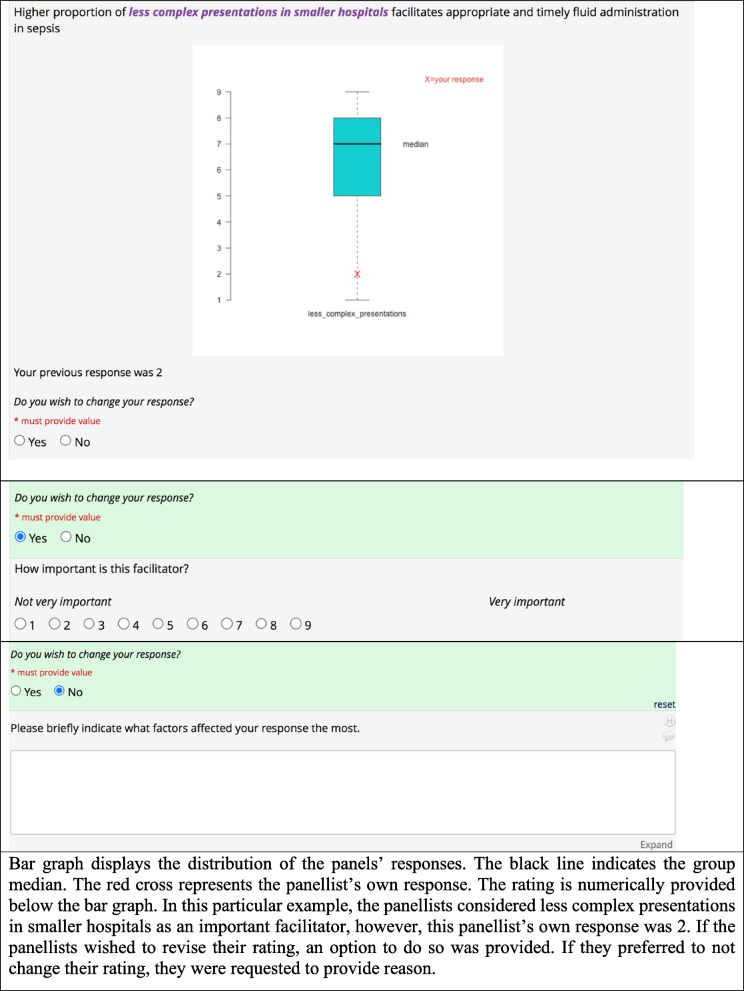
Round 2 discussion and feedback

 In Round 2, panellists were provided access to view anonymised comments obtained from Round 1. Panellists then reviewed bar charts showing their own responses in relation to the group’s distribution of Round 1 responses (Fig. 2). Below each bar graph, we displayed options for panellists to either revise their rating or not to change their rating, and to provide rationale for their choice. The free texts with rationales for their rating enabled exploration of the reasons for disagreement among the panellists.

### Data analysis

The R statistical language software (version 4.0.3, R Foundation for Statistical Computing, Vienna, Austria) was used for analysis of data from both modified Delphi rounds.

#### Round 1

The modified Delphi survey data were analysed descriptively. The widely used RAM approach [10] was used to determine the ratings level. Ratings between (1-3) were considered as low ratings; (4-6) as uncertain ratings and (7-9) as high ratings. Bar charts were created for each panellist, illustrating their response to each of the statements. The bar graph (Fig. 2) represents a distribution of panellists’ responses along with an indicator for the group median. The red cross displays a panellist’s own response. The qualitative comments from Round 1 were summarised using a summary table. The comments were not altered (except for editorial changes) and the wordings used by the panellists were provided in Round 2 in accordance with the Delphi survey guidelines [9]. If the panellist’s individual response fell outside of the group ratings (low, uncertain or high ratings in relation to the group median), the panellists were provided the option to revise or provide comment on the reason for their choice of ratings in Round 2.

The texts from Rounds 1 and 2 were analysed using directed content analysis [17] with pre-determined deductive parent themes (i.e., (i) barriers, (ii) facilitators and (iii) strategies) generated from the previous phases of the study. The key verbatim extracts have been presented as meaning units and then condensed into condensed meaning units through open coding, categorization and abstraction to align with the parent themes that are also part of the Delphi survey. To better understand the perspectives of panellists who wished to retain their Round 1 rating, exemplar statements of Round 2 comments are provided in Appendix 2.

#### Round 2

We determined the perceived importance of each barrier, facilitator, and strategy by the key stakeholders panel, using a 2-step RAM approach [10]. Firstly, new medians for panel ratings and measures of dispersion were calculated using Round 2 data from the 18 panellists. The agreement level was calculated using the definition of agreement specified in RAM approach. Accordingly, as this study had a panel size of 18 which meets the multiples of three criteria by RAM, disagreement was defined as “the minimum number of panellists permitted to rate outside the region containing the median must be one less than the number of panellists rating in the extremes of disagreement.” [10] Therefore, in this study, if a statement had more than five panellists ratings within the region containing the median, it was considered as “in agreement”. The qualitative comments provided by panellists for their choice of ratings have been summarised (Appendix 2).

## Results

Table 1 shows the demographic characteristics of Round 1 panellists. Panellists were recruited from all four participating sites and had nearly equal number of nurses and physicians.

**Table 1:** Characteristics of Round 1 panellists

Role of the Panellist	No of panellists in the role	Number of years of ED experience
Senior Staff Specialist	8	10–30
Nurse Consultant	3	8–35
Medical Head of Department	2	23–25
Nurse Manager	2	8–23
Nurse Educator	2	5–7
Nurse Unit Manager	4	9–20
Total	21	5–35

Of the 21 panellists, the years of emergency department work experience ranged from 5 to 35 years. Several participants held direct administrative and managerial responsibilities and others held educational and supervision responsibilities.

**Fig. 3:**
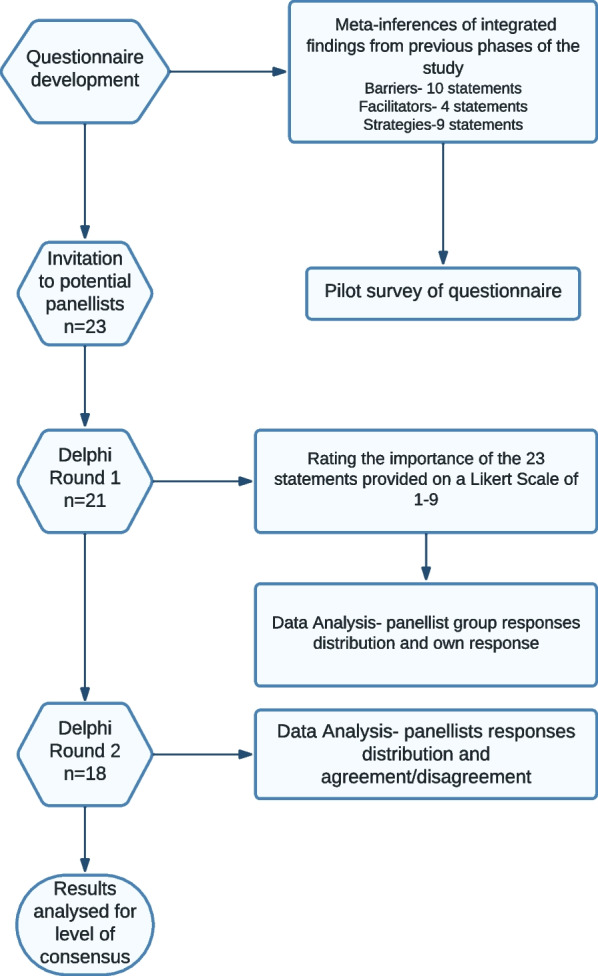
Flow Chart for the modified delphi study process

 Figure 3 shows the flow of the modified Delphi process. Out of the 23 potential panellists invited, 21 completed Round 1 survey and 18 (86%) completed both Round 1 and Round 2 surveys.

**Fig. 4:**
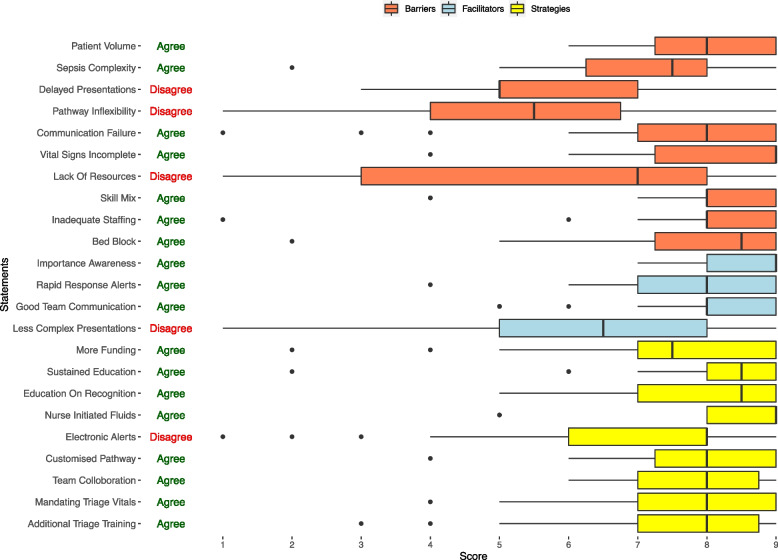
Level of consensus regarding the importance attributed to the barriers, facilitators, and strategies

 Out of the 10 statements provided as barriers, the panellists rated 9 (90%) statements as important; of the 4 facilitator statements, 3 (75%) were rated important; and all the 9 (100%) strategy statements were rated as important (Fig. 4). One barrier and one facilitator statement received an uncertain rating (Median ratings 4–6) [10]. The statements receiving the highest median ratings were: Incomplete vital signs at triage (Median = 9, IQR 7.25 to 9.00) a barrier; awareness of importance of fluid administration in sepsis (Median = 9, IQR 8.00 to 9.00) a facilitator; and provision of nurse-initiated intravenous fluids in sepsis (Median = 9, IQR 8.00 to 9.00) a strategy to improve practice. Figure 4 illustrates the ratings for the 23 statements included in the survey.

There was agreement with 18 (78%) of the statements. Panellists showed disagreement regarding (i) delayed presentations to ED, sepsis pathway inflexibility, lack of resources as barriers; (ii) less complex presentations to smaller hospitals as facilitator and (iii) use of more intelligent electronic alerts as an improvement strategy. The results of the directed content analysis are presented in Table 2. The results provide insight into the contextual reasons why the panellists expressed disagreement with five of the statements.

**Table 2:** Directed content analysis of comments from panellists (disagreement rationale)

Theme	Meaning Unit	Condensed meaning unit	Category
***Barriers***	Delayed presentation ought not be a factor in appropriate and timely fluid administration. Though I recognise access block is root cause, most crumbly or older patients probably get a bed fairly early, yet they seem not to get fluid.	Sicker patients from delayed presentations are recognised early but may not receive fluids due to individual characteristics.	Delayed presentation
	Clinicians tend to ‘customise’ care anyway or vary treatment based on the context. This is already happening I suspect. I believe the main barrier is the lack of consensus on when and how much fluid to give in sepsis. Time to antibiotics is clearer but time and amount of fluid required is not. There is confusion around fluid resuscitation in sepsis and when to introduce inotropes. I think all of this gives mixed message to clinicians	Clinical judgement supersedes sepsis guidelines in practice.	Pathway inflexibility
	Mismatch of resources at different times is issue. I’ve waited an hour for triage and five doctors free. Never really experienced a lack of equipment	Mismatch of resources and variability between settings and professions accounts for the difference.	Lack of resources
***Facilitators***	I feel that if you have less complex patients within the department, as an ED clinician, it is quite easy to leave that patient and prioritise a patient that is unstable. Often, I find the issue is that there is a large volume of unwell patients, and then the demand is higher, making it difficult to prioritise between competing demands. Apart from trauma, we see (i.e., a smaller ED) very complex patients. Ultimately after initial assessment and treatment we then transfer these complex patients out - they still present to the ED. I do not agree - working in smaller hospitals definitely noted better patient care values.	Variations in hospital settings rather than patient’s characteristics facilitate fluid administration.	Less complex presentations
***Strategies***	Alert overload, I don’t even read them anymore Do not agree - instructions still need to be carried out seamlessly with less roadblocks/steps.	Alert fatigue and poor clinical usefulness impede use of electronic alerts.	Electronic alerts

## Discussion

The aim of this study as part of a larger mixed-method study was to identify the level of consensus among key emergency stakeholders who were the modified Delphi panellists regarding the factors identified [5] and reported by emergency clinicians [6] in the previous phases of the study as barriers, facilitators, and strategies to improve appropriate and timely administration of initial intravenous fluids in patients with sepsis in the emergency department. Using the RAM approach, our study highlights that most of the determinants that the emergency clinicians considered as barriers, facilitators, and strategies to improve fluid administration in sepsis were deemed as important and in addition, most of the factors were agreed upon by the stakeholders currently in key roles of management, leadership, supervision, and education in the emergency departments. None of the 23 factors identified by the clinicians in the previous phases [5, 6] received overall lower ratings among the key stakeholders.

Our study shows some uncertainty in the level of importance for three factors and some disagreement among the stakeholders for five factors. However, factors with either uncertain importance level ratings or disagreement should not be automatically treated as unimportant determinants. They need to be interpreted in the context of the panellists’ work setting. For instance, some factors that were considered significant in larger hospitals may not be applicable to smaller hospitals. The panellists have provided rationales for their ratings where they have disagreed with the rest of the panel and have also expressed some concerns over strategies such as electronic alerts. Reviewing the potential concerns raised by the panellists (Table 2 and Appendix 2), will help determine how and what concerns need to be addressed while these factors are incorporated into future implementation strategies.

The following factors were rated the highest level of importance (median score of 9) (i) Incomplete set of vital signs at triage as a barrier; (ii) awareness of importance of fluid administration as a facilitator and (iii) nurse-initiated intravenous fluids as a strategy. These three factors all relate to clinician factors rather than patient related factors.

Incomplete vital signs at triage, specifically related to sepsis is an under researched area. While triage decisions are shown not to be influenced by the acquisition of a patient’s vital signs [18], incomplete triage has been found to be associated with adverse effects for patients including increased mortality [19–21]. Although a few panellists expressed concerns about the delay that mandating vital signs at triage may have on the time to complete the triage process, most agree that the benefits would far outweigh the risks. This consequently led panellists to rate and agree with mandating the completion of a complete set of vital signs at triage as an important strategy to improve fluid administration. This concurs with the findings from a previous study [22] where 100% completion of triage vital signs data improved the compliance with fluid administration in sepsis.

Awareness of the importance of intravenous fluid administration in sepsis was rated as a very important facilitator to timely intravenous fluid administration. This has been a proven facilitator with the recent mass campaigns on sepsis management by the Surviving Sepsis guidelines which has resulted in a considerable reduction in the mortality rate over several decades [3]. However, panellists agreed that maintaining sustained education on the recognition of sepsis among emergency clinicians is an essential strategy to improve timely fluid administration.

The nurse-initiated intravenous fluid strategy was rated and agreed to as a highly important strategy to improve timely intravenous fluid management in sepsis. While the study by Bruce et al. [23] showed a significant improvement with overall sepsis management associated with a nurse-initiated sepsis bundle, the administration of intravenous fluid was not independently nurse-initiated in the study, which most likely led to the suboptimal compliance with intravenous fluid administration. Our study supports independent nurse-initiated intravenous fluid administration, with support from key managers and leaders including heads of departments, senior staff specialists, nurse unit managers and educators as a strategy to significantly improve timely intravenous fluid administration. Nevertheless, some caution was expressed by a few of the key stakeholders (Appendix 2) associated with potential challenges. A few stakeholders were concerned about the accurate identification by nurses of patients who require volume fluid resuscitation and recognition of any associated risk-factors such as co-morbidities for rapid infusion should be considered while implementing nurse-initiated fluids. Strategies such as further education, and additional triage training as agreed to by the panellists would negate the risk of harm associated with the clinically inappropriate initiation of intravenous fluids.

Panellists rated and agreed with the identified barriers related to the lack of human resources, such as staff shortages and skill mix, and non-human factors such as bed block, as important. The lack of non-human resources such as equipment for cannulation, IV fluid administration pumps, IV poles and giving sets was identified as a barrier, and received a higher rating overall, but there was disagreement between the panellists. The disagreement may have been attributed to the differences between the four hospital settings which included two small and two large metropolitan hospitals. There were differences reported in the clinical responsibilities between nurses and physicians while administering intravenous fluids. Nurses routinely source the equipment for tasks such as IV cannulation and administering intravenous fluids. The high rating is congruent with a previous study reporting a lack of non-human resources in overcrowded emergency departments as a barrier to timely patient treatment [24]. The increased patient volume has resulted in ongoing emergency department overcrowding and in bed or access block on a routine basis. Most panellists rated and agreed that bed block is a very important barrier. The impact of overcrowding and access block on patient outcomes such as increased mortality is well documented in the literature [25] and the findings in this study echoes those concerns.

The last decade saw initiatives such as the 4-hour rule in Australian emergency departments, which for a short-term, reduced the burden of overcrowding [26]. Improvement in patient outcomes and improved mortality associated with reduction in patient volume as a result of these initiatives have also been documented [27]. However, more recent data suggests the inability to sustain these short-term initiatives because of the growing population demands, and the declining number of publicly available beds. In Australia, hospitals have reduced beds from 2.65 to 1000 population in 2010–2012 to 2.53 per 1000 population in 2018–2019. Over the same period, the number of patients visiting the emergency department has increased by 25% (an increase of over 737,400 patients) causing bed blocks on a routine basis [28, 29]. Panellists confirmed that bed block has a direct impact on time-to treatment interventions; also supported by a recent systematic review by Darraj et al. [30] and intravenous fluid administration in sepsis is one such time-critical intervention.

While the inflexibility of the current Sepsis Pathway used in the study sites has been suggested as a barrier to intravenous fluid administration, it was rated as uncertain with disagreement. The idea of having a customised Sepsis Pathway with scope for including clinical judgement, appealed to the key stakeholders who provided a high importance rating and agreed upon this strategy. The perception of inflexibility of the pathway as a barrier, was substantiated by comments from the panellists about how clinical judgement supersedes the pathway and therefore, adherence or even use of the pathway in its current form is not common practice. This finding supports the consideration for including nurse-initiated intravenous fluids and the ability to customise fluid volume based on individual patient needs in a modified Sepsis Pathway. In addition, intelligent electronic alerts incorporated into the Sepsis Pathway could be developed as adjuncts and accommodate the concerns of the key stakeholders, such as avoiding “alert fatigue.”

In summary, this study supports the findings from previous studies reporting strategies like electronic alerts, and additional sepsis education for nurses and physicians in emergency departments to improve intravenous fluid management in sepsis [22, 31–34]. More importantly, this study provides new understanding of the factors that are specifically related to the study sites, for instance, team communication barriers, rapid response alerts facilitating prompt action, and strong support for the provision of nurse-initiated intravenous fluids in sepsis.

### Strengths and limitations of the study

To the best of the author’s knowledge, this study is the first Delphi study seeking to identify the level of consensus regarding barriers, facilitators, and strategies specifically to improve appropriate and timely administration of intravenous fluids in sepsis in emergency departments. The statements used to develop the questionnaire were those directly provided by clinicians involved in direct patient care, working in the same study sites obtained in the previous phases of the study. The perceived factors were rated, and the majority obtained consensus from the key stakeholders such as heads of departments, senior staff specialists, nurse unit managers and educators from the same study sites. The key strength of the study is obtaining consensual findings from the key stakeholders or leaders who have a direct influence on implementation. In line with the CFIR guidelines [6], anything less than wholehearted support from leaders, will doom implementation of change to failure. Obtaining consensus is therefore critical which was the aim of this study.

Our study has some limitations. The questionnaire was pilot tested for content structure, readability, and navigation to ensure transferability [9]. However, it was not assessed for validity because we used the findings from the previous phases of the study to develop the questionnaire. The study was conducted across four hospitals within a similar geographical context using the same protocols, with some of the key stakeholders, especially the senior staff specialists working across the four settings, and therefore generalisation of these results to a different setting may be limited. There is no universally accepted method of achieving consensus and the number of rounds used to include in a Delphi [9, 35]. To overcome this, we have used the widely acclaimed RAM approach in determining consensus and agreement. While our study offers consensual findings on strategies to be implemented, specific details of the intervention need to be further discussed with the key stakeholders and this could vary from one hospital to another.

## Conclusion

We have identified 18 factors attaining modified Delphi panel consensus associated with appropriate and timely administration of intravenous fluids in sepsis in the emergency departments. Although five factors were rated as uncertain, they offer valuable insight into what factors need to be considered while designing interventions. The findings from this study have face validity as they involve experts with extensive experience in managing sepsis patients in emergency departments. The study contributes to the body of Australian research on important facilitators, barriers, and strategies to improve fluid administration in sepsis. It reaffirms the perceived importance of key barriers such as lack of human and non-human resources, in addition to locally specific barriers like incomplete triage vital signs and provides insight into the support for actionable strategies such as nurse-initiated intravenous fluids and customised Sepsis Pathway. Further exploration on operationalising these strategies can optimise the effectiveness of future intravenous fluid administration practices translating into better patient outcomes.

### Supplementary Information

**Additional file 1: Appendix 1.** Delphi Survey Questionnaire: Emergency Department (ED)nurses and doctors

**Additional file 2: Appendix 2. **Summary table of exemplar comments from panellists in Round 2

## Data Availability

The data analysed in this study are not publicly available due to ethical restrictions.
