# Supplementary material for: Facilitators and barriers of appropriate and timely initiation of intravenous fluids in patients with sepsis in emergency departments: a consensus development Delphi study
Source: BMC Nurs. 2023 Oct 27;22:402. doi: 10.1186/s12912-023-01561-w (PMC10604401; doi:10.1186/s12912-023-01561-w)
Supplement: Supplementary file 1 — Additional file 1: Appendix 1. Delphi Survey Questionnaire: Emergency Department (ED)nurses and doctors [file 12912_2023_1561_MOESM1_ESM.docx]

**Appendix 1**

**Delphi Survey Questionnaire: Emergency Department (ED)nurses and doctors -**

Facilitators and barriers of appropriate and timely initiation of initial fluid therapy in patients presenting with sepsis in the emergency department

| **Your Details** |
| --- |

**1) How many years have you worked in an ED?**

**2) Please indicate your position/s held**

Staff Specialist

ED Consultant

Nurse Manager

Nurse Unit Manager

Nurse Educator

Nurse Consultant

**3) Which area best describes your role/s in the ED?**

Education

Management

Leadership

Supervision

*The statements used in this survey are derived from the findings from previous retrospective chart reviews and focus groups among ED nurses and doctors.*

***Section 1:*** *Evaluation of barriers, facilitators, and intervention strategies*

| **Please rank the statements below in order of importance from your perspective. On a scale of 1-9, where 1 means “Not very important” and 9 means “Very important”, please rank each statement.** |
| --- |

| **Barriers of appropriate and timely fluid administration in sepsis in ED** |
| --- |

- *The higher volume of patients presenting to ED impacts appropriate and timely fluid administration in sepsis*

How important is this barrier?

**1 2 3 4 5 6 7 8 9**

Not very important Very important

- *The complexity of sepsis patients presenting to ED impacts appropriate and timely fluid administration in sepsis*

How important is this barrier?

**1 2 3 4 5 6 7 8 9**

Not very important Very important

- *Delayed presentations (presenting at later stages of sepsis) due to lower health literacy of patients impacts appropriate and timely fluid administration in sepsis*

How important is this barrier?

**1 2 3 4 5 6 7 8 9**

Not very important Very important

- *Sepsis pathway is not commonly used due to its inflexibility and lack of room for clinical judgement*

How important is this barrier?

**1 2 3 4 5 6 7 8 9**

Not very important Very important

- *Failure to communicate effectively as a team of doctors and nurses impacts appropriate and timely fluid administration in sepsis*

How important is this barrier?

**1 2 3 4 5 6 7 8 9**

Not very important Very important

- *Lack of complete set of vital signs at triage results in flawed/incomplete triage assessment and impacts timely and accurate recognition of sepsis*

*How important is this barrier?*

**1 2 3 4 5 6 7 8 9**

Not very important Very important

- *Lack of resources such as cannulation equipment, IV poles etc. impacts timely fluid administration in sepsis*

How important is this barrier?

**1 2 3 4 5 6 7 8 9**

Not very important Very important

- *Staffing skill mix issues (junior heavy) e.g., inexperience with assessment, cannulation etc. impacts appropriate and timely fluid administration in sepsis*

How important is this barrier?

**1 2 3 4 5 6 7 8 9**

Not very important Very important

- *Inadequate staffing (nurses and doctors) impacts appropriate and timely fluid administration in sepsis*

How important is this barrier?

**1 2 3 4 5 6 7 8 9**

Not very important Very important

- *Bed blocks/access blocks impacts appropriate and timely fluid administration in sepsis*

How important is this barrier?

**1 2 3 4 5 6 7 8 9**

Not very important Very important

| **Facilitators of appropriate and timely fluid administration in sepsis in ED** |
| --- |

- *Generally, nurses and doctors are aware of the importance of initial fluid resuscitation in sepsis*

How important is this facilitator?

**1 2 3 4 5 6 7 8 9**

Not very important Very important

- *Escalation of care using Rapid response alerts (Yellow zone/Red Zone criteria) facilitates appropriate and timely fluid administration in sepsis*

How important is this facilitator?

**1 2 3 4 5 6 7 8 9**

Not very important Very important

- *Relatively good team communication in smaller hospitals (nurses and doctors aware of team members) facilitates appropriate and timely fluid administration in sepsis*

How important is this facilitator?

**1 2 3 4 5 6 7 8 9**

Not very important Very important

- *Higher proportion of less complex presentations in smaller hospitals facilitates appropriate and timely fluid administration in sepsis*

How important is this facilitator?

**1 2 3 4 5 6 7 8 9**

Not very important Very important

| **Strategies to improve appropriate and timely initial fluid administration in sepsis** |
| --- |

- *More funding to improve staffing and resources like equipment will improve appropriate and timely initial fluid administration in sepsis*

How important is this strategy?

**1 2 3 4 5 6 7 8 9**

Not very important Very important

- *Education of ED nurses and doctors to sustain knowledge about fluid administration in sepsis will improve appropriate and timely initial fluid administration in sepsis*

How important is this strategy?

**1 2 3 4 5 6 7 8 9**

Not very important Very important

- *Education of ED nurses and doctors to improve knowledge about recognition of sepsis will improve appropriate and timely initial fluid administration in sepsis*

How important is this strategy?

**1 2 3 4 5 6 7 8 9**

Not very important Very important

- *Provision for advanced practice such as nurse-initiated fluids (Normal Saline bolus) in the Sepsis Pathway will improve appropriate and timely initial fluid administration in sepsis*

How important is this strategy?

**1 2 3 4 5 6 7 8 9**

Not very important Very important

- *More intelligent and integrated electronic alerts with the ability to send reminders for fluid administration for example based on patient factors such as vital signs will improve appropriate and timely initial fluid administration in sepsis*

How important is this strategy?

**1 2 3 4 5 6 7 8 9**

Not very important Very important

- *Redesigning the sepsis pathway with the provision to customise based on patient characteristics such as advanced age, fluid restriction, presenting features etc. will improve appropriate and timely initial fluid administration in sepsis*

How important is this strategy?

**1 2 3 4 5 6 7 8 9**

Not very important Very important

- *Team building and collaboration across different heirarchial levels will improve appropriate and timely initial fluid administration in sepsis*

How important is this strategy?

**1 2 3 4 5 6 7 8 9**

Not very important Very important

- *Mandating a complete set of vital signs at triage for all suspected sepsis patients will improve appropriate and timely initial fluid administration in sepsis*

How important is this strategy?

**1 2 3 4 5 6 7 8 9**

Not very important Very important

- *Additional triage training to recognise sepsis will improve appropriate and timely initial fluid administration in sepsis*

How important is this strategy?

**1 2 3 4 5 6 7 8 9**

Not very important Very important

***Section 2:*** *Panellist’s input for barriers, facilitators and strategies*

1) From your perspective, please describe possible barriers (not included above) preventing appropriate and timely administration of initial fluids in sepsis (word limit 25 words)

|  |
| --- |

2) From your perspective, please describe possible facilitators (not included above) that will support appropriate and timely administration of initial fluids in sepsis (word limit 25 words)

|  |
| --- |

3) From your perspective, please describe likely interventions (not included above) that will support appropriate and timely administration of initial fluids in sepsis (word limit 25 words)

|  |
| --- |
