# Supplementary material for: Facilitators and barriers of appropriate and timely initiation of intravenous fluids in patients with sepsis in emergency departments: a consensus development Delphi study
Source: BMC Nurs. 2023 Oct 27;22:402. doi: 10.1186/s12912-023-01561-w (PMC10604401; doi:10.1186/s12912-023-01561-w)
Supplement: Supplementary file 2 — Additional file 2: Appendix 2. Summary table of exemplar comments from panellists in Round 2 [file 12912_2023_1561_MOESM2_ESM.docx]

| **Appendix 2** Summary table of exemplar comments from panellists in Round 2 |
| --- |
| **Barriers**  Patient volume-  *Those patients with severe sepsis often have protocol commencement performed early now with current models of care. Issue is probably more around patient selection as those patients that are hypotensive will often be rapidly escalated and have fluid administered quite early*  Sepsis complexity-  *Agree that patients are complex. However, sepsis treatment should be the same across the board, albeit lower thresholds for therapy e.g., fluids, inotropy etc*  *Volume of patients is causing more delays than complexity*  Lack of resources-  *I don't think this is an actual barrier*  *Never really experience a lack of equipment*  Delayed presentations-  Delayed presentation ought not be a factor in appropriate and timely fluid administration.  Sepsis Pathway inflexibility-  *Clinicians tend to 'customise' care anyway or vary treatment based on the context. This is already happening I suspect.*  *Patients with septic shock are often*  *- recognised early*  *- treated early*  *Those patients with occult forms of sepsis with high morbidity have medical conditions that might preclude the use of sepsis pathways (CCF, transplant, immunosuppression, renal failure etc) makes these protocols extremely unnecessary* |
| **Facilitators**  Rapid response alerts-  *In clinical practice I have seen a poor response to Rapid Response alerts where there is failure to review the patient at the bedside.*  Less complex patients-  *I feel that if you have less complex patients within the department, as an ED clinician, it is quite easy to leave that patient and prioritise a patient that is unstable. Often, I find the issue is that there are a large volume of unwell patients, and then the demand is higher, making it difficult to prioritise between competing demands.*  *Apart from trauma, we see (i.e., a smaller ED) very complex patients. Ultimately after initial assessment and treatment we then transfer these complex patients out - they still present to the ED.*  *Working in a small hospital, definitely noted better patient care values* |
| **Strategies**  More funding-  *It will have some limited impact*  *More funding for staffing/resources should not be considered until confirmation the existing staffing and resources are fully utilised at optimal performance. There is a disproportionate amount of underperforming staff in EDs collectively impacting overall performance.*  More intelligent electronic alerts-  *Instructions still need to be carried out seamlessly with less roadblocks/steps*  *Alert overload, I don't even read them anymore*  Additional Triage training-  *There is a lot of focus on triage already, where we know there is a huge cognitive, emotional and physical burden. Additional training should happen downstream*  Sustained education-  *Reinforcement of sepsis pathway required - I believe sepsis is already taught at all levels of medicine/nursing, including pre-registration.*  *The educated/senior staff are unable to lead by example - which in my opinion is the best education for the rest*  Mandating triage vitals-  *Mandated for "all" suspected sepsis patients will steal away treatment time while the full set of vitals are being done.*  Nurse-initiated fluids-  *Do not agree it’s going to make a difference and is not going to be associated with adverse events*  *Improper identification has the potential of more harm in a large cohort of patients* |
